# Poor glycemic control impairs oral health in children with type 1 diabetes mellitus - a systematic review and meta-analysis

**DOI:** 10.1186/s12903-024-04516-y

**Published:** 2024-06-28

**Authors:** Zsuzsanna Triebl, Bulcsú Bencze, Dorottya Bányai, Noémi Rózsa, Péter Hermann, Dániel Végh

**Affiliations:** 1 Diabetes-Dental Workgroup, Semmelweis University, Szentkirályi 47, Budapest, 1088 Hungary; 2 Department of Paediatric Dentistry and Orthodontics, Semmelweis University, Szentkirályi 47, Budapest, 1088 Hungary; 3 Department of Prosthodontics, Semmelweis University, Szentkirályi 47, Budapest, 1088 Hungary

**Keywords:** Type 1 diabetes mellitus, Children, DMFT, Salivary flow rate, Periodontal health

## Abstract

**Objective:**

There are more than one million children and adolescents living with type 1 diabetes mellitus, and their number is steadily increasing. Diabetes affects oral health through numerous channels, including hyposalivation, immune suppression, and the inflammatory effect of glycation end-products. However, patients with type 1 diabetes must follow a strict sugar free diet that is proven to be carioprotective. Therefore, the aim of this systematic review and meta-analysis is to investigate whether children with type 1 diabetes have a difference in Decayed, Missing, Filled Teeth index (DMFT), salivary function, and periodontal status than children without diabetes, with an emphasis on glycemic control.

**Materials and Methods:**

PubMed, Embase and Cochrane libraries were screened for articles, using predefined search keys without any language or date restrictions. Two independent authors performed the selection procedure, extracted data from the eligible articles, carried out a manual search of the reference lists, and assessed the risk of bias using the Newcastle-Ottawa scale. Meta-analysis was performed in R using the random-effects model. Effect sizes were mean differences; subgroup analysis was performed on glycemic control.

**Results:**

33 studies satisfied the eligibility criteria. 22 studies did not show a significant difference regarding the DMFT index between the diabetes and non-diabetes groups; six studies found that children living with diabetes had higher DMFT scores, compared to five studies that found significantly lower scores. Meta-analysis found no statistically significant differences in plaque, gingival, and calculus indexes, however it found significant differences in pooled DMFT indexes, and salivary flow rate. Subgroup analysis on glycemic control using DMFT values found significant differences in children with good and poor glycemic control with results of 0.26 (CI95%=-0.50; 1.03) and 1.46 (CI95%=0.57; 2.35), respectively.

**Conclusions:**

Children with poor glycemic control face higher risk of developing caries compared to good control and non-diabetes children. Regular dental check-ups and strict control of glycemic levels are highly advised for children living with type 1 diabetes, further emphasizing the importance of cooperation between dentists and diabetologists.

## Introduction

Diabetes Mellitus (DM) is a disorder that is caused by either the lack of insulin secretion or the insufficient effect of the hormone [1], that leads to a chronically increased blood glucose level, which harms human health in several ways [2].

DM has four main types: type 1 is caused by an autoimmune response against the beta-cells of the pancreas; type 2 can develop on a multifactorial basis, mainly by an unhealthy lifestyle with the addition of bad diet and obesity; gestational diabetes develops and usually recedes within the gestational period; and lastly secondary diabetes that is either caused by certain medications or other illnesses [3]. There is still some uncertainty on the exact reason behind the development of type 1 DM; numerous causes are mentioned in the current literature including genetic (HLA proteins) and nongenetic factors (viral infections such as Coxsackievirus B) [4, 5].

It was estimated that the number of people affected by DM to be at 536,3 million in 2021, and projected to reach 783 million by 2045 [6]. A significant portion of the affected individuals consists of children and adolescents and approximately 1.2 million of them have type 1 DM [6]. According to Chobot et al., the incidence of type 1 DM increased from 5.36 to 22.74 per 100 000 capita in 24 years’ time [7]. Several studies showed that there is a consistent increase in the number of affected children, approximately 3%, per year [8].

Hyperglycemia is the main cause of the clinical symptoms: elevated blood sugar levels can cause polyuria, weight loss despite heightened appetite, blurred vision, excessive thirst, constant tiredness and diabetic ketoacidosis [9]. Diagnosis relies on symptoms alongside an oral glucose tolerance test (OGTT), although evaluating metabolic control can also be achieved through measuring the HbA1c level; furthermore, the presence of autoantibodies associated with diabetes can be examined [10].

Dental caries is widespread all around the world [11]. Facilitated by biofilms and various factors, leads to localized demineralization of teeth [12]. Additionally, there were studies that reported on the harmful effects of DM on oral health, namely higher caries rate in children with type 1 DM, significantly higher plaque accumulation, gingivitis and calculus deposition [13–15].

According to Nederfors, salivary dysfunctions can be classified into three main groups: xerostomia, hyposalivation and changes in the composition of saliva [16]. Xerostomia is known to be the subjective complaint of oral dryness [17], whereas hyposalivation means the decrease in salivary outflow, that can be objectively measured [18]. Hyposalivation can go together with xerostomia, but that’s not always the case – on the other hand, sometimes xerostomia is present without real salivary gland dysfunction [19].

DM is considered to cause lower salivary flow rate [2], which can also induce harmful complications such as caries [20] and oral candidiasis [21]. Hyposalivation, poor immune defense, and high blood sugar levels are the main risk factors of developing oral candidiasis [21, 22]. A suppressed immune system does not only make the human body susceptible to infections [22], but it also has a negative effect on wound healing [23].

DM has a bidirectional relationship with periodontal health, namely because DM promotes periodontal inflammation through various pathophysiological pathways that influence immune cells, collagen and lipid metabolism [11, 12, 24], while periodontitis can have serious adverse effects on glycemic control [25]. High blood sugar levels can lead to the formation of advanced glycation end-products, which enhance the production of inflammatory cytokines. In this manner the speed of periodontal bone resorption increases rapidly [26].

There is still debate on the overall effect of type 1 DM on oral health; on one hand, lower salivary functions and higher salivary glucose levels shift the oral environment towards a more cariogenic milieu, on the other hand patients with DM should follow a strict sugar-free diet, that has a serious carioprotective effect [27]. The relationship between type 2 DM and oral health is more certain, however, the impact of type 1 DM is still contradictory. There is data in the literature that type 1 DM decreases [28], or has no significant effect on caries prevalence [29], and also that it increases calculus and gingival indices [30].

There is no previous analysis in the literature that investigates the effect of different glycemic controls on oral health in children with type 1 DM. Therefore, we decided to investigate the effect of type 1 DM and glycemic control on caries prevalence, salivary flow rate and periodontal indices.

## Materials and methods

This review was created according to the standards of the PRISMA® (Preferred Reporting Items for Systematic Reviews and Meta-Analyses) Statement. The PICO (P, population/patient/problem; I, intervention; C, comparison; O, outcome) question we investigated in this review was formed according to the rules of PRISMA®:

“Do children (P) living with Type 1 Diabetes Mellitus (I), compared to healthy children (C), have worse caries and periodontal indexes? (O)

The protocol of the review was preregistered on PROSPERO (CRD42023449223).

### Inclusion and exclusion criteria

Studies were included, if they (1) were cross-sectional and case-control studies; (2) included patients under the age of 19; (3) included only type 1 DM. Studies were excluded if they (1) did not report on any of the predefined outcomes; (2) were about other fields of dentistry; (3) were animal studies; (4) were inadequate article types, such as notes, reviews, letters, conference abstracts or randomized controlled studies; (5) had high risk of bias.

### Information sources, search strategy and the selection process

An extensive search strategy was employed to identify eligible studies through the following electronic databases: Pubmed, Cochrane Library, and Embase. The complete search key used was the following: ((diabetes OR DM OR diabetes mellitus OR diabetic) AND (type 1 OR type-1 OR type one OR insulin dependent OR IDDM)) AND (children OR child) AND (caries OR decay OR oral health status OR DMF OR gingival index OR calculus index OR salivary flow rate OR plaque index). The keywords were linked with the help of Boolean operators. The databases were screened on May 30, 2024.

The results were exported to Endnote [31]. After duplicate removal, which was done with the help of the automatic duplicate finder in Endnote, two calibrated independent authors searched for articles according to the predefined inclusion and exclusion criteria with the help of Rayyan.ai [32], where the title and abstract selection was conducted. Disagreements were solved by consensus. If no consensus was achieved a third reviewer helped with the decision. The final pool of included studies was decided upon completing the full-text selection procedure under similar conditions. Agreements between the reviewers were calculated by Cohen’s Kappa. A manual search of the included papers reference list was conducted using the online Citation chaser tool [33].

### Quality assessment and data extraction

The quality assessment of the included studies was done by the same two independent reviewers based on the guidelines of the Newcastle-Ottawa scale for case-control and cross-sectional studies.

Two authors have extracted the necessary data independently using Excel (Microsoft) forms. The following data were extracted: first, the year the article was published; second, the names of the authors; and third, the title of the study. The number and type of different case and control groups were recorded, the parameters they examined, the number of the examined children in their respective groups, ages, and sex distributions were recorded. Data on Decayed, Missing due to caries, and Filled Teeth (DMFT) index (categorical outcomes) and the parameters of the saliva, including salivary flow rate (continuous outcomes) and the quantity of the saliva (continuous outcomes were recorded). Some studies recorded the results of the Oral Hygiene Index-Simplified (OHI-S), the Plaque Index (PI) (Silness-Löe), the Calculus Index (CI) (Greene and Vermilion), and the Gingival Index (GI) (Löe-Silness) which were also extracted.

The results and conclusions of each study were summarized to make the comparison more easily manageable and the results straightforwardly accessible.

### Publication bias and certainty of evidence

Publication bias was assessed by funnel plots when at least 10 studies were available.

Certainty of evidence was assessed by one reviewer with the Grading of Recommendations Assessment, Development, and Evaluation (GRADE) tool.

### Data Synthesis and Analysis

For the analysis, a random-effects model was chosen based on the assumption of significant between-study heterogeneity. The predefined included outcomes were all continuous, therefore the effect size measure was the difference between the means (MD) with 95% CI. A result that didn’t contain the null value was considered statistically significant. Subgroup analysis was performed based on the glycemic control of the patients; differentiation was made between well-, and poorly controlled patients based on their HbA1c values; for standardization purposes patients with lower than 7,5–8% HbA1c were allocated to the well-controlled, and higher than 7,5–8% were allocated to the poorly controlled group. Furthermore, between-study heterogeneity was calculated with the I2 statistics. Descriptive statistics were used to show the results of the meta-analysis with forest plots. Subgroup analyses were performed using the glycemic control data of the patient groups. All statistical analyses were carried out with R (version 4.3.0) using the meta (version 6.2.1) package for basic meta-analysis calculations and plots.

## Results

### Result of the systematic search and quality assessment

**Fig. 1:**
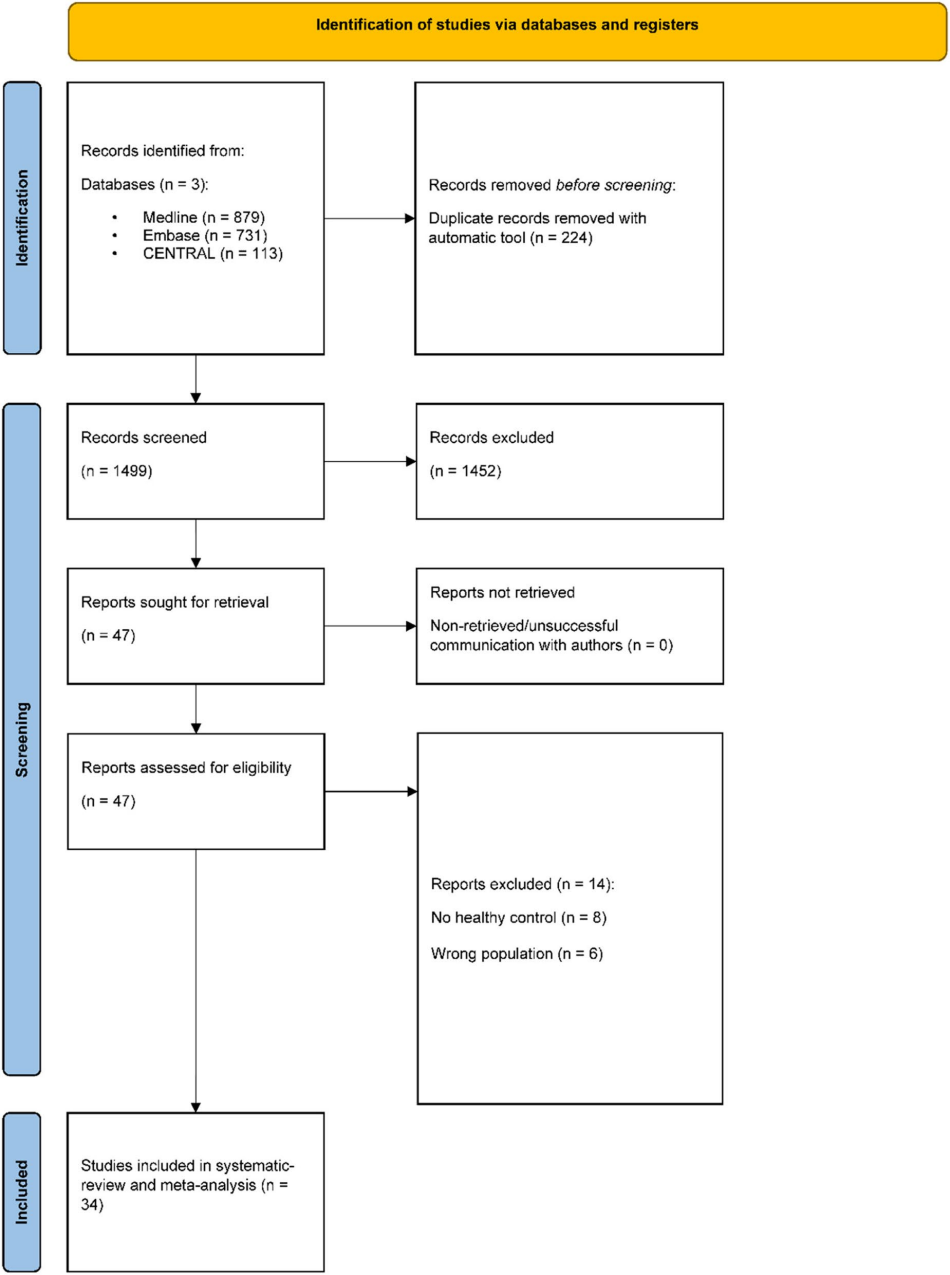
Prisma flowchart (2020), detailed explanation of the selection procedure

From the systematic search 1723 articles were retrieved, after the duplication removal 1499 articles were assessed by title and abstract selection (κ = 0.81). Conducting the full text selection, 34 eligible articles were identified for further analysis (κ = 1). The databases were screened on May 30, 2024. No additional eligible studies were found at the manual searches of the reference lists. The detailed selection procedure can be found in Fig. 1.

For the included studies it was required to have transparent inclusion and exclusion criteria, measurements of outcomes, adequate statistical analysis and consistent reporting of outcomes. To increase the certainty of the evidence, studies with low to moderate risk of bias (above a score of five) were included, whereas studies with high risk (below a score of five) were excluded from further analysis. The risk of bias assessment of studies is shown in Table 1.

**Table 1:** Risk of Bias assessment of the included studies using the Newcastle-Ottawa Scale for case-control, cross-sectional studies

First author, year	Selection				Comparability of cases and controls	Outcome			Total score
	Adequate case definition	Representativeness of cases	Selection of controls	Definition of controls		Ascertainment of exposure	Same method of ascertainmen	Nonresponse rate	
Akpata, 2006, Kuwait [14]	*	*	*	*	*	*	*	*	8
Alves, 2012, Brazil [55]	*	*	*	-	*	-	*	*	6
Al-Badr, A. H., 2021, Saudi Arabia [43]	*	*	*	-	*	-	*	*	6
Amer, 2019, Iraq [56]	*	*	-	*	*	-	-	*	5
Arheiam, 2014, Libya [57]	*	*	*	*	*	*	*	*	8
Assiri, S. A, 2022, Saudi Arabia [44]	*	*	*	*	*	*	*	*	8
Babatzia, A., 2019, Greece [47]	*	*	-	*	*	*	*	*	8
Babu, K. L. G., 2018, India [35]	*	*	*	*	*	*	*	*	8
Banyai, D., 2022, Hungary [64]	*	*	*	*	*	*	*	*	8
Bassir, 2014, Iran [41]	*	*	*	*	*	*	*	*	8
Coelho, Asedc, 2018, Portugal [51]	*	*	-	*	*	-	*	*	6
Djuričkovic, M., 2021, Montenegro [52]	*	*	*	*	*	*	*	*	8
Elheeny, A. A. H., 2020, Egypt [45]	*	*	*	*	*	*	*	*	8
El-Tekeya, 2012, Egypt [46]	*	*	*	*	*	*	*	*	8
Ferizi, L., 2018, Kosovo[53]	*	*	-	*	*	-	*	*	6
Geetha, S., 2019, India [36]	*	*	*	*	*	*	*	*	8
Goteiner, 1986, The United States [49]	*	*	*	*	-	*	*	*	7
Govindaraju, 2024, India [38]	*	*	*	*	*	-	*	*	7
Iscan, T. A., 2020, Turkey [54]	*	*	-	*	-	*	*	*	6
Ismail, A. F., 2017, Hong Kong [61]	*	*	*	*	*	*	*	*	8
Kamran, S., 2019, Iran [27]	*	*	*	*	*	*	*	*	8
Lai, S., 2017, Italy [60]	*	*	*	*	*	-	*	*	7
Manjushree, R., 2022, India [37]	*	*	*	*	*	*	*	*	8
Matsson, 1975, Sweden [58]	*	*	*	*	*	-	*	*	7
MesaroS, A., 2019, Romania [59]	*	*	*	*	*	-	*	*	7
Pachonski, M., 2020, Poland [50]	*	*	*	*	*	*	*	*	8
Pappa, E., 2020, Greece [48]	*	*	*	*	*	*	*	*	8
Rafatjou, 2016, Iran [42]	*	*	*	*	*	*	*	*	8
Rai, 2011, India [39]	-	*	*	*	-	-	-	*	5
Sadeghi, R., 2017, Iran [40]	*	*	*	*	*	*	*	*	8
Al-Mutairi, 2020, Saudi Arabia [34]	-	*	*	-	-	-	-	*	3
Siudikiene J, 2006, Lithuania [63]	*	*	*	*	*	*	*	*	8
Swanljung O, 1992, Finland [62]	*	*	*	*	*	*	*	*	8
Tagelsir, 2010, Belgium [29]	*	*	*	*	*	*	*	*	8

The study of Al-Mutari et al. has received high risk of bias due to the contradictions in the abstract and in the full text of the article. They had conflicting outcomes in the Results section compared to the conclusion in the main text [34].

### General aspects of the included studies

All in all, the included articles were from 14 countries. There were five studies from India [35–39], four from Iran [27, 40–42],two from Saudi Arabia [43, 44], two from Egypt [45, 46], two from Greece [47, 48], one from Kuwait [14], one from The United States [49] one from Poland [50], one from Portugal [51], one from Montenegro [52], one from Kosovo [53], one from Turkey [54], one from Brazil [55], one from Iraq [56], one from Libya [57], one from Sweden [58], one from Belgium [29], one from Romania [59], one from Italy [60], one from Hong Kong [61], one from Finland [62], one from Lithuania [63] and one from Hungary [64].

The youngest child in the cohort was two-year-olds, while the oldest one was eighteen years old. Altogether, 5048 children were examined: 2547 children living with type 1 DM and 2501 non-DM children.

The included articles analyzed the oral health of children with DM in comparison with their sex and age-matched controls without DM. The parameters under investigation included the following: DMFT, DMFS (Decayed, Missing due to caries, and Filled Surface), dmft (decayed, missing, and filled primary teeth) indexes, ICDAS (International Caries Detection and Assessment System), stimulated or unstimulated salivary flow rate, buffer capacity, viscosity and glucose level of the saliva, CI, PI, GI (Table 2).

**Table 2:** Basic characteristics and examined parameters

Author, year, country	Study design	DM group	Control group	Age of participants	Caries outcomes	*p*-value (DMFT)	Salivary outcomes	Periodontal outcomes
Akpata, 2006, Kuwait [14]	cross-sectional study	*n* = 53	*n* = 53	12–15 years-olds	DMFT: statistically significantly higher in DM	< .04	salivary flow rate: no statistically significant difference buffering capacity: no statistically significant difference	
Alves, 2012, Brazil	cross-sectional study	*n* = 51 female: 22 male: 29	*n* = 51 female: 32 male: 19	6–18 years-olds	DMFT: no statistically significant difference deft: no statistically significant difference	DMFT: 0.43 deft: 0.14		
Al-Badr, A. H., 2021, Saudi Arabia [43]	cross-sectional study	*n* = 69 female: 37 male: 32	*n* = 140 females: 80 males: 60	6–12 years-olds	DFT: no statistically significant difference between the groups	0.681		
Amer, 2019, Iraq [56]	cross-sectional study	*n* = 40	*n* = 40	10–13 years-olds	DMFT: statistically significantly higher in DM	0.001		
Arheiam, 2014, Libya [57]	cross-sectional study	*n* = 70 female: 25 male: 45	*n* = 70 female: 25 male: 45	10–15 years-olds	DMFT: no statistically significant difference	0.071		
Assiri, S. A, 2022, Saudi Arabia [44]	case-control study	*n* = 40 females: 22 males: 18	*n* = 40 females: 22 males: 19	6–12 years-olds	DMFT: no statistically significant difference dmft: statistically significantly lower in DM	DMFT: 0.145; dmft: 0.019	salivary flow rate: no statistically significant difference buffering capacity: statistically significantly lower in DM	
Babatzia, A., 2019, Greece [47]	cross-sectional study	*n* = 74 females: 43 males: 31	*n* = 70 females: 41 males: 29	6–15 years olds	DMFS: no statistically significant difference	> 0.05	salivary flow rate: normal buffering capacity: high buffering capacity in all children	GI: no statistically significant difference CI: no statistically significant difference
Babu, K. L. G., 2018, India [35]	cross-sectional study	*n* = 80 females: 38 males: 42	*n* = 80 females: 34 males: 46	6–18 years old	deft: lower in DM, but no statistically significant difference DMFT: statistically significantly higher in DM	DMFT: 0.009; deft: 0.073		GI: no statistically significant difference
Banyai, D., 2022, Hungary [64]	cross-sectional study	*n* = 200 females: 104 males: 96	*n* = 173 females: 109 males: 64	3–18 years old	DMFT: no statistically significant difference	0.054897; 0.074063		
Bassir, 2014, Iran [41]	cross-sectional study	*n* = 31 female: 18 male: 13	*n* = 31 female: 18 male: 13	7–17 years-olds	DMFT: no statistically significant difference	0.33		
Coelho, Asedc, 2018, Portugal [51]	cross-sectional study	*n* = 36 females: 18 males: 18	*n* = 36 females: 18 males: 19	mean age of 13 years and four months	DMFT: no statistically significant difference	> 0.05	salivary flow rate: no statistically significant difference	CI: statistically significantly higher in DM PI: statistically significantly higher in DM
Djuričkovic, M., 2021, Montenegro [52]	cross-sectional study	*n* = 87 females: 39 males: 48	*n* = 90 females: 43 males: 47	10–15 years olds	DMFT: no statistically significant difference	0.866	salivary flow rate: statistically significantly lower in DM buffering capacity: high in DM	
Elheeny, A. A. H., 2020, Egypt [45]	case-control study	*n* = 222 female: 116 male: 106	*n* = 222 female: 104 male: 118	8–14 years old	DMFT: statistically significantly higher in DM	0.0001		GI: statistically significantly higher in DM PI: statistically significantly higher in DM
El-Tekeya, 2012, Egypt [46]	case-control study	*n* = 50 female: 29 male: 21	*n* = 50 female: 22 male: 28	6–9 years-olds	DMFS: no statistically significant difference	0.85		PI: statistically significantly higher in DM GI: statistically significantly higher in DM
Ferizi, L., 2018, Kosovo [53]	case-control study	*n* = 80	*n* = 80	10–15 years old	DMFT: statistically significantly higher in DM	0.001	buffering capacity: statistically significantly lower in DM salivary flow rate: statistically significantly lower in DM	
Geetha, S., 2019, India [36]	case-control study	*n* = 175	*n* = 175	10–15 years-old	DMFT: statistically significantly lower in DM	0.008		CI: statistically significantly higher in DM
Goteiner, 1986, The United States [49]	cross-sectional study	*n* = 169 female: 72 male: 97	*n* = 80 female: 40 male: 40	5–18 years-olds	DMF: no statistically significant difference			GI: no statistically significant difference
Govindaraju, 2024, India [38]	case-control study	*n* = 66 female: 36 male: 30	*n* = 66 female: 36 male: 30	4–10 years-olds	DMFT: no statistically significant difference	0.504		GI: no statistically significant difference
Iscan, T. A., 2020, Turkey [54]	case-control study	*n* = 50 females: 26 males: 24	*n* = 50 females: 20 males: 30	6–13 years old	DMFT: no statistically significant difference dmft: lower in DM, but no statistically significant difference severe caries: statistically significantly more in controls	DMFT: 0.447; dmfs: 0.288		PI: statistically significantly lower in DM
Ismail, A. F., 2017, Hong Kong [61]	case-control study	*n* = 32 females: 16 males: 16	*n* = 32 females: 16 males: 17	mean age: 12 ± 4 years old	DMFt: no statistically significant difference dmft: no statistically significant difference	DMFT: 0.44; dmft: 0.66		GI: no statistically significant difference CI: no statistically significant difference
Kamran, S., 2019, Iran [27]	cross-sectional study	*n* = 100 females: 57 males: 43	*n* = 100 females: 57 males: 43	9–14 years old	DMFT: no statistically significant difference	0.654		
Lai, S., 2017, Italy [60]	case control study	*n* = 68 females: 35 males: 33	*n* = 136 females: 70 males: 66	4–14 years old	ICDAS: no statistically significant difference	> 0.05		
Manjushree, R., 2022, India [37]	cross-sectional study	*n* = 40	*n* = 40	12–16 years old	DMFT: statistically significantly lower in DM	> 0.05	salivary flow rate: statistically significantly lower in DM	PI: statistically significantly lower in DM
Matsson, 1975, Sweden [58]	cross-sectional study	*n* = 33	*n* = 33	9–16 years-olds	DFS: significantly lower in DM	0.01		
MesaroS, A., 2019, Romania [59]	case-control study	*n* = 15	*n* = 15	5–17 years old	DMFT: similar in DM and non-DM	-		
Pachonski, M., 2020, Poland [50]	case-control study	*n* = 50 females: 24 males: 26	*n* = 25 females: 13 males: 12	10–18 years old	DMFT: no statistically significant difference	0.18		GI: no statistically significant difference PI: no statistically significant difference
Pappa, E., 2020, Greece [48]	cross-sectional study	*n* = 100 females: 60 males: 40	*n* = 50 females: 30 males: 20	10–18 years old	DMFT: no statistically significant difference	> 0.05	buffering capacity: no statistically significant difference salivary flow rate: no statistically significant difference	
Rafatjou, 2016, Iran [42]	case-control study	*n* = 80 female: 46 male: 34	*n* = 80 female: 46 male: 34	5–18 years-olds	DMFT: no statistically significant difference	0.158		PI: no statistically significant difference GI: statistically significantly higher in DM
Rai, 2011, India [39]	cross-sectional study	*n* = 100	*n* = 100	6–12 years-olds	DMFT: statistically significantly lower in DM	0.0005	salivary flow rate: statistically significantly lower in DM	
Sadeghi, R., 2017, Iran [40]	cross-sectional study	*n* = 50 females: 34 males: 16	*n* = 50 females: 34 males: 16	6–12 and 13–18 years old	DMFT: statistically significantly higher in DM	0.08; 0,05		GI: statistically significantly higher in DM CI: no statistically significant difference PI: statistically significantly higher in DM
Siudikiene J, 2006, Lithuania [63]	cross-sectional study	*n* = 68	*n* = 68	10–15 years-olds	DMFS: statistically significantly lower in DM			
Swanljung O, 1992, Finland [62]	cross-sectional study	*n* = 85 female: 46 male: 39	*n* = 85 female: 46 male: 39	12–18 years-olds	DMF: no statistically significant difference DMFS: no statistically significant difference		buffering capacity: no statistically significant difference salivary flow rate: no statistically significant difference	
Tagelsir, 2010, Belgium [29]	cross-sectional study	*n* = 52 female: 23 male: 29	*n* = 50 female: 22 male: 28	2–16 years-olds	DMFT: no statistically significant difference dmft: no statistically significant difference	DMFT: 0.35 dmft: 0.27		

*Abbreviations*: *DM* Diabetes Mellitus, *DMFT* Decayed, Missing due to caries, and Filled Teeth, *DMFS* Decayed, Missing due to caries, and Filled Surface, *GI* Gingival Index, *CI* Calculus Index, *PI* Plaque Index, *ICDAS* International Caries Detection and Assessment System

### Glycemic control

Several articles differentiated between the quality of glycemic control. Ten study divided the DM study group into further groups according to their metabolic control [27, 29, 40, 46–48, 50, 59, 60, 63]; five articles defined good glycemic control (GGC) and poor glycemic control (PGC) [47, 48, 50, 60, 63]. Whereas five articles included a third group called intermediate glycemic control (IGC) [27, 40, 46, 29, 59]. The HbA1c values used to define the sub-groups are shown in Table 3.

**Table 3:** The connections between DMFT index and the salivary flow rate

	Flow rate is significantly lower *n* = 5	Flow rate, no significant difference *n* = 5	Normal flow rate *n* = 1
DMFT is significantly higher	5/1 20%	5/1 20%	
DMFT no significant difference	5/2 40%	5/4 80%	1/1 100%
DMFT is significantly lower	5/2 40%		

*Abbreviations*: *DMFT* Decayed, Missing due to caries, and Filled Teeth

Seven articles examined the buffer capacity in relation to the prevalence of caries [14, 44, 47, 48, 52, 53, 63], two reported significantly worse buffer capacity in children living with DM [43, 53], and one of these two have reported significantly higher scores on DMFT index [53]. From the three article reporting no significant differences between the study and the control group with respect to buffer capacity, two did not find a significant difference concerning the DMFT index either [48, 63] and one found significantly higher DMFT [14]. Two articles have reported higher buffer capacity, though not significantly higher values, while there was no significant difference between the DMFT indexes either [47, 52] (Table 4).

**Table 4:** The connections between the DMFT index and the buffer capacity of the saliva

	Buffer capacity is significantly lower *n* = 2	Buffer capacity no significant difference *n* = 3	High buffer capacity *n* = 2
DMFT is significantly higher	2/1 50%	3/1 33%	
DMFT no significant difference	2/1 50%	3/2 66%	2/2 100%
DMFT is significantly lower			

*Abbreviations*:*DMFT* Decayed, Missing due to caries, and Filled Teeth

### Caries indexes

The included studies exhibited a high degree of heterogeneity with respect to the analysis of DMFT index, which stands for the number of decayed, missing due to caries, and filled teeth [65].

Twenty-two studies did not find statistically significant differences between the study group and the control group [27, 29, 38, 41–44, 46–52, 54, 55, 57, 59–62, 64]. There were six studies revealing higher DMFT values in the DM groups [14, 35, 40, 45, 53, 56]; and five studies found that children living with type 1 DM had lower DMFT values, which means a better caries prevalence [36, 37, 39, 58, 63].

All those studies that found the DMFT index significantly worse revealed poorer results in many other aspects, such as higher PI and GI [45], lower buffer capacity and salivary flow rate [53].

Interestingly, the study conducted by Elheeny et al. reported higher DMFT index in the DM group, even though they brushed their teeth significantly more [45]. Babu et al. reported that the DMFT index was higher in children with DM, however their GI was comparable [35]. The study of Geetha et al. disclosed that the DMFT index in children with DM was significantly lower, while their CI were significantly higher [36]. One other study stated that the study group had better DMFS and PI indexes despite having a lower salivary flow rate and a higher salivary glucose level [37].

All the other studies revealed that there was no statistically significant difference between the study and control groups regarding the DMFT or DMFS indexes. From these 22 articles, twelve showed a higher DMFT value in DM groups, but these differences were not significant [27, 38, 44, 46, 29, 42, 49, 51, 55, 57, 62, 64], and there were five studies in which children with DM had better DMFT values than healthy controls [41, 43, 52, 54, 61]. The remaining five articles did not report on the comparison of healthy and DM individuals, only comparing the groups divided by metabolic control [47, 48, 50, 59, 60].

**Fig. 2:**
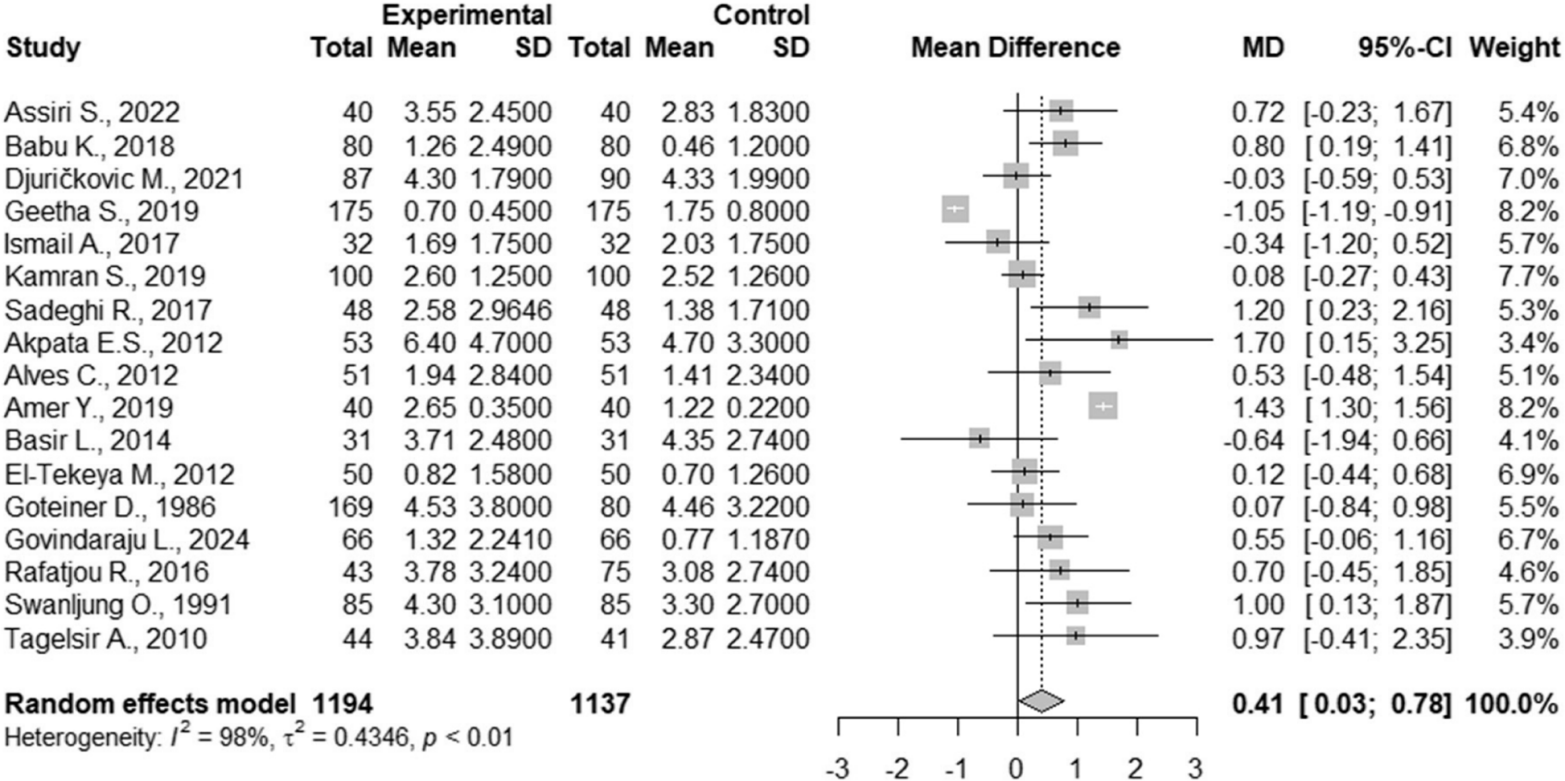
Meta-analysis of the pooled DMFT values compared in children with and without DM

There were 17 studies included in the meta-analysis [14, 27, 29, 35, 36, 38, 40–42, 44, 46, 49, 52, 55, 56, 61, 62]. Statistically significant differences were found between the groups, with a result of 0.41 (CI95%=0.03; 0.78). The between study heterogeneity was considered very high and significant I2=98% (Fig. 2).

After dividing children living with DM into groups according to their metabolic control, there were a few articles that did not find statistically significant differences between the groups [27, 40, 46, 47, 29, 59, 60]. Three articles found significant differences between different metabolic controls [48, 50, 63]. Pachonski et al. reported that there was a significant difference between children with PGC and GGC regarding the DMFT index, and children with GGC had the best DMFT values among the groups, including the healthy controls, while children with PGC had the worst values [50]. According to the study of Pappa et al., even though there was no significant difference between children with GGC and no DM in terms of DMFT, there was a significant difference between the GGC and PGC groups and a significant difference between the PGC and control group [48]. Babatzia reported that children with PGC had higher DMFS values, although not significant [47]. According to the study of Siudikiene, children living with DM had significantly lower DMFS score compared to non-DM children, patients with well-controlled DM had significantly less decayed surface, to poorly controlled individuals [63].

**Fig. 3:**
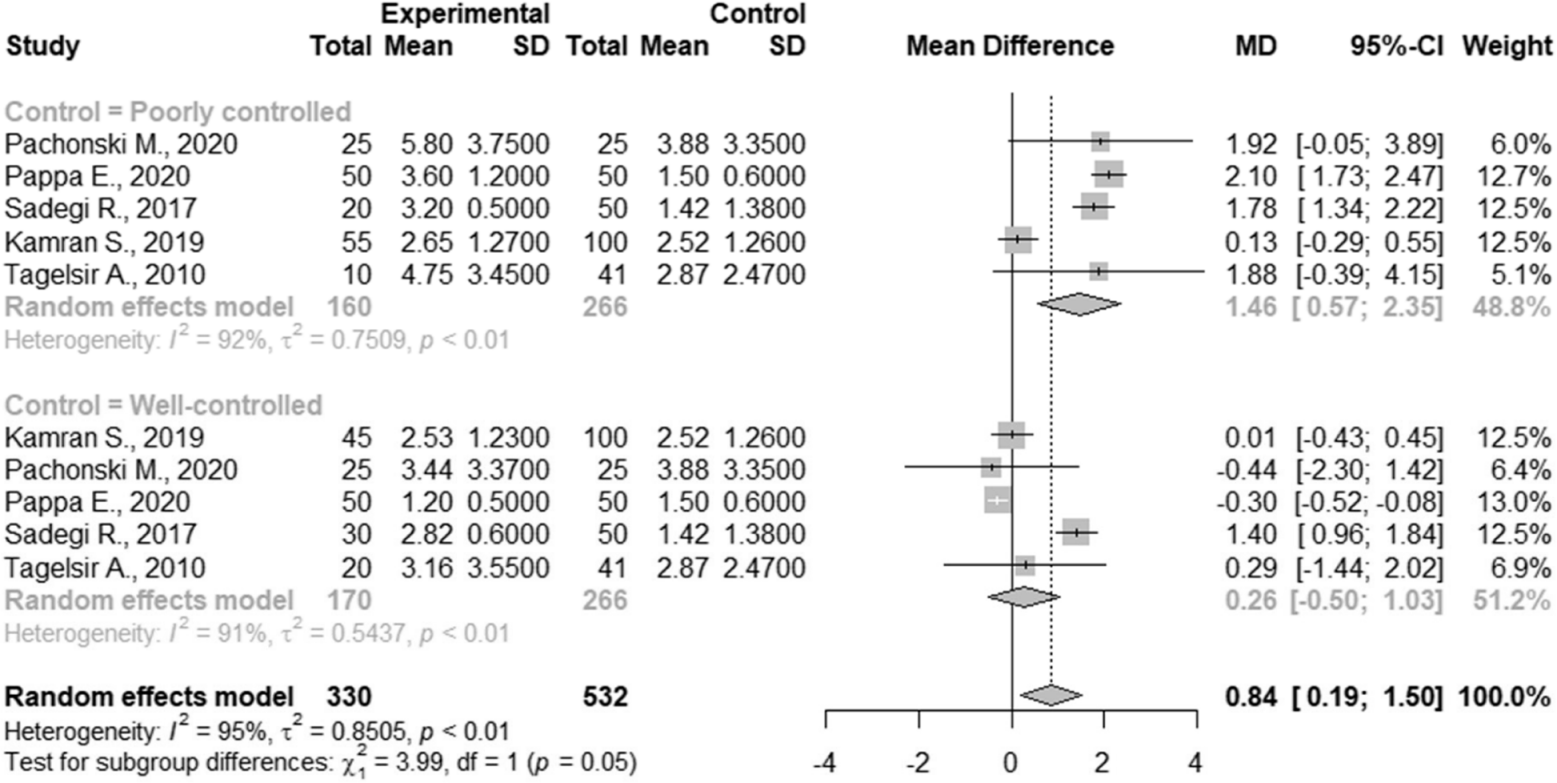
Subgroup Meta-analysis of DMFT index in well- and poorly controlled children compared with children without DM

There were five studies included in the meta-analysis of DMFT with subgroup analysis based on their glycemic control [27, 29, 40, 48, 50]. There was a statistically significant difference between poorly controlled patients and non-DM patients with a result of 1.46 (CI95%=0.57; 2.35). The between study heterogeneity was considered very high and statistically significant I2=92%; there was no difference between the well-controlled and non-DM patients (Fig. 3).

### Salivary parameters

Seven articles investigated the buffer capacity of children with DM [14, 44, 47, 48, 52, 53, 62]. Two articles showed statistically significantly worse buffer capacity [44, 53], three articles did not find significant differences between the study and control group [14, 48, 62], and two studies reported better results in the DM group, while the buffer capacity of these children was not significantly higher compared to children without DM [47, 52].

Eleven study examined salivary flow rate, from which five studies examined stimulated salivary flow rate [44, 47, 52, 53, 62], four study examined the unstimulated flow rate [37, 39, 51, 55], and two examining both the stimulated and the resting salivary flow rate [14, 48]. Five of them have reported significantly worse results [37, 39, 52, 53, 55], five studies revealed no significant difference between the study and control groups [14, 44, 48, 51, 62], and lastly, one study reported comparable outcomes in children with DM to non-DM children [47].

Out of the three articles where they found the flow rate significantly worse in the study group than in the control group [37, 39, 52, 53, 55], there was one article that reported significantly higher DMFT scores [53], two with no significant difference [52, 55], and two with significantly lower DMFT index [37, 39]; whereas the five articles where they found no significant difference in the salivary flow rate, four of them also showed no significant difference in the DMFT scores [44, 48, 51, 62], except for the study of Akpata, where the DMFT index was significantly higher in DM children [14] (Table 3).

Seven articles examined the buffer capacity in relation to the prevalence of caries [14, 44, 47, 48, 52, 53, 62], two reported significantly worse buffer capacity in children living with DM [43, 53], and one of these two have reported significantly higher scores on DMFT index [53]. From the three article reporting no significant differences between the study and the control group with respect to buffer capacity, two did not find a significant difference concerning the DMFT index either [48, 62] and one found significantly higher DMFT [14]. Two articles have reported higher buffer capacity, though not significantly higher values, while there was no significant difference between the DMFT indexes either [47, 52] (Table 4).

**Fig. 4:**
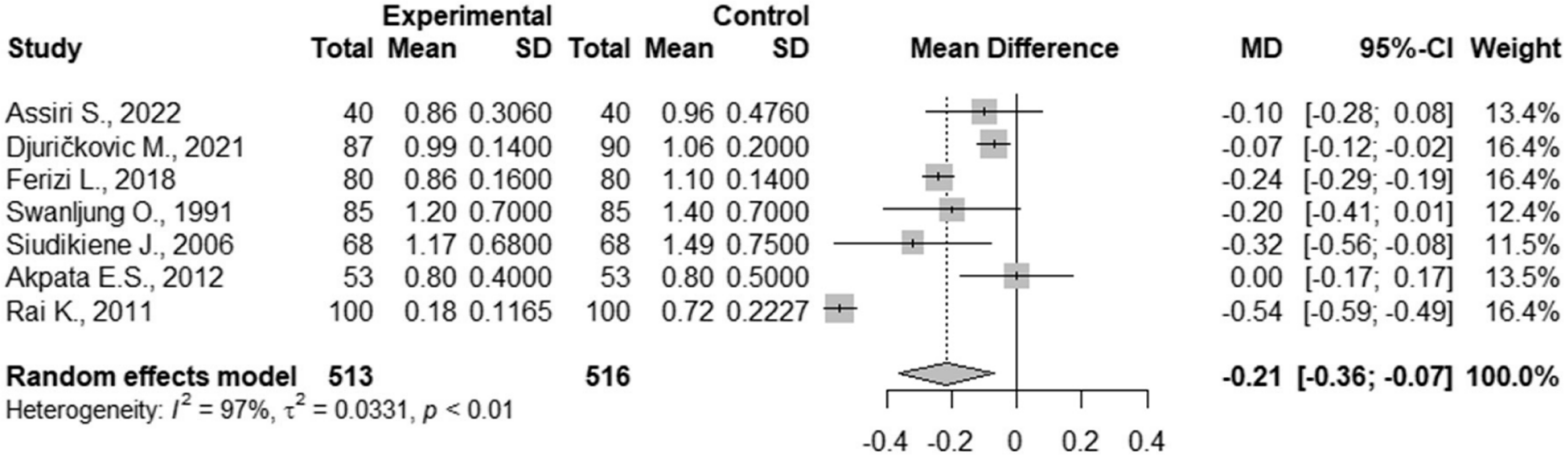
Meta-analysis of stimulated salivary flow rate compared in children with and without DM

There were seven studies included in the meta-analysis of salivary flow rate [14, 44, 52, 53, 62, 63]. There were statistically significant differences between the groups with a result of -0.21 (CI95%=-0.36; -0,07). The between study heterogeneity was considered very high and significant I2=97% (Fig. 4).

Only three of the seven articles recorded data about metabolic control and salivary parameters. Pappa et al. reported that salivary flow rate and pH values were significantly lower in the PGC group than in the GGC group and controls [48], while others found that the flow rate of all children was normal with sufficient capacity [47]. Siudikiene et al. found that there were no significant differences between the groups in terms of salivary flow rate and buffering capacity [63].

### Periodontal indexes

Considering periodontal indexes, GI, PI, and CI were examined.

There were nine studies reporting on GI scores. Four articles showed higher GI scores in children living with DM [40, 42, 45, 46], and five articles did not find significant differences [35, 38, 47, 50, 61]. There were no data about significantly better GI scores; however, in one study the gingival conditions of DM children were considered healthy [35].

**Fig. 5:**
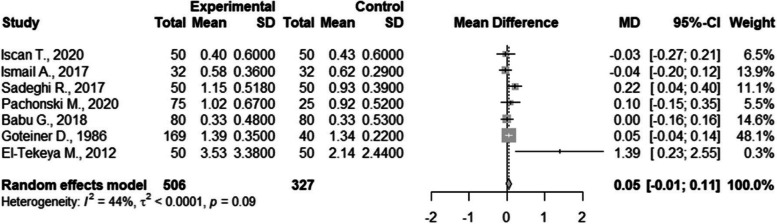
Meta-analysis of Löé & Silness gingival index values compared in children with and without DM

Seven studies were included in the quantitative analysis of GI that was comparable and used the Löe and Silness index [35, 39, 40, 46, 49, 50, 54, 61]. There were no statistically significant differences between the groups with a result of 0.05 (CI95%=-0.01; 0.11). The between study heterogeneity was considered low and statistically non-significant I2=44% (Fig. 5).

Regarding CI, two out of five studies have reported significantly higher scores in children living with DM [36, 51], and three did not find statistically significant differences between the groups [40, 47, 61]. Just as in the case of GI scores, there was not a significantly better CI score recorded in the DM group.

**Fig. 6:**
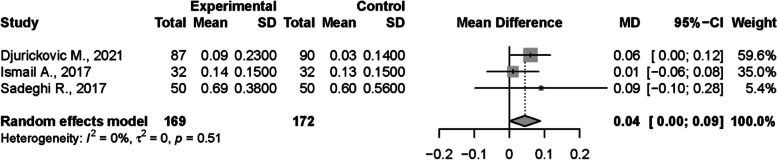
Meta-analysis of Greene and Vermilion calculus index values compared in children with and without DM

Meta-analysis was conducted on three studies regarding CI that used Greene and Vermilion indexes [40, 52, 61]. There were no statistically significant differences between the groups with a result of 0,04 (CI95%=-0,00; 0,09). The between study heterogeneity was considered very low and non-significant I2=0% (Fig. 6).

Nine articles reported on PI, from which five articles found significantly higher PI scores in the DM group [40, 45, 46, 51]. Among these four articles, one applied this observation only to children with poor metabolic control [47]. There were two studies with non-significant differences between the groups [42, 50], while two studies have reported lower PI scores in the DM group [37, 54].

**Fig. 7:**
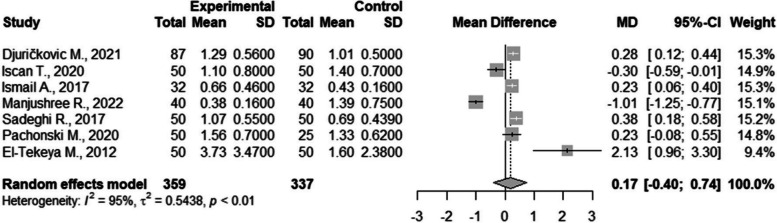
Meta-analysis of Silness & Löé plaque index values compared in children with and without DM

There were seven studies included in the meta-analysis of PI [37, 40, 46, 50, 52, 54, 61]. There were no statistically significant differences between the groups with a result of 0.17 (CI95%=-0.40; 0.74). The between study heterogeneity was considered very high and statistically significant I2=95% (Fig. 7).

Three studies that examined DM children according to different metabolic controls did not find significant differences between the groups regarding the conditions of the periodontium and oral hygiene (PI, GI, and CI) [40, 46, 50]. Even though Babatzia et al. have reported that there was no significant difference between GI and CI scores, they found that children with PGC had significantly more dental plaque [47].

### Publication bias and certainty of evidence

**Fig. 8:**
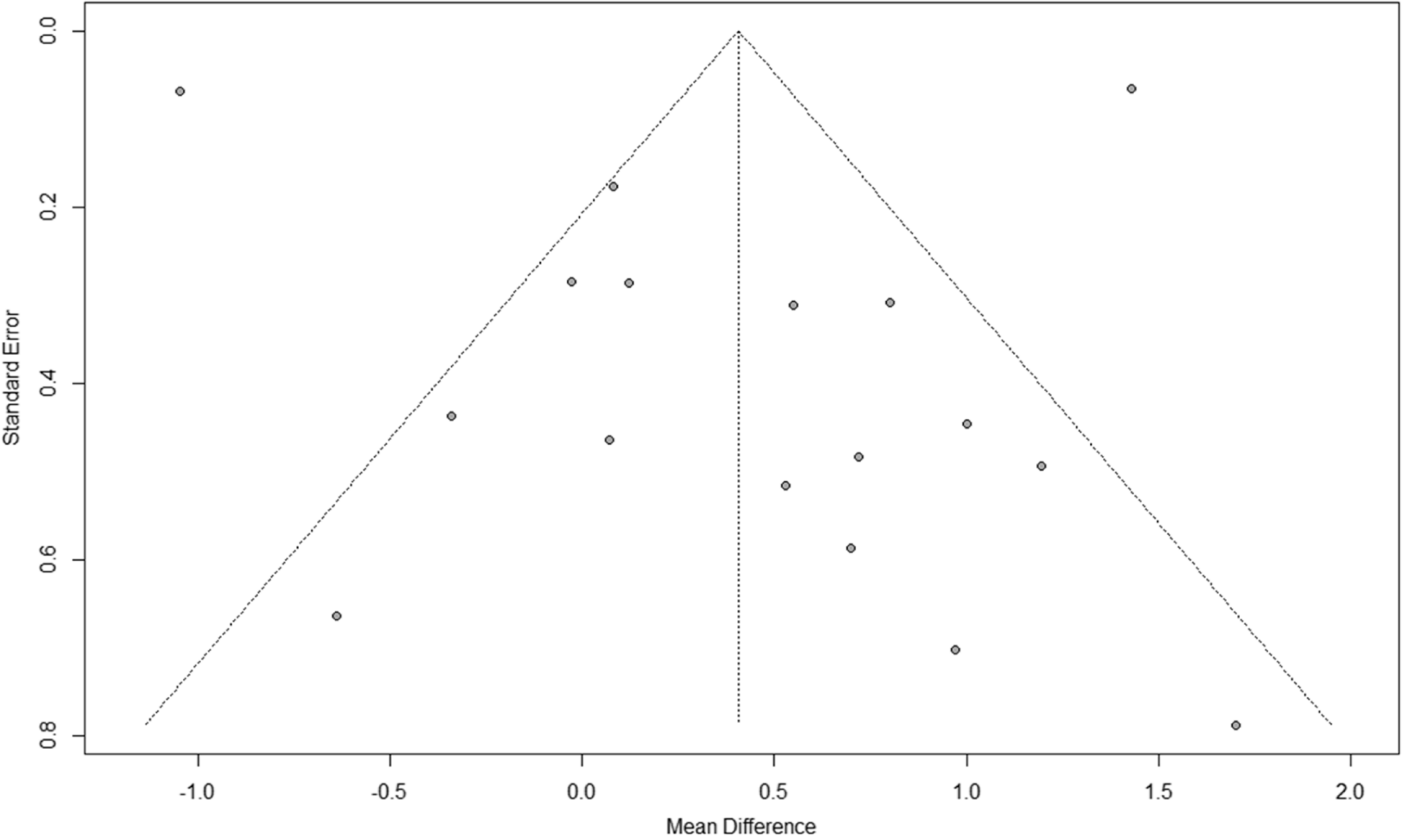
Funnel plotof publication bias in DMFT outcomes

With analyses containing at least 10 studies, publication bias was assessed by generating funnel plots. DMFT outcomes have provided symmetrical funnel plots, hence the probability of the existence of publication bias is low (Fig. 8).

**Fig. 9:**
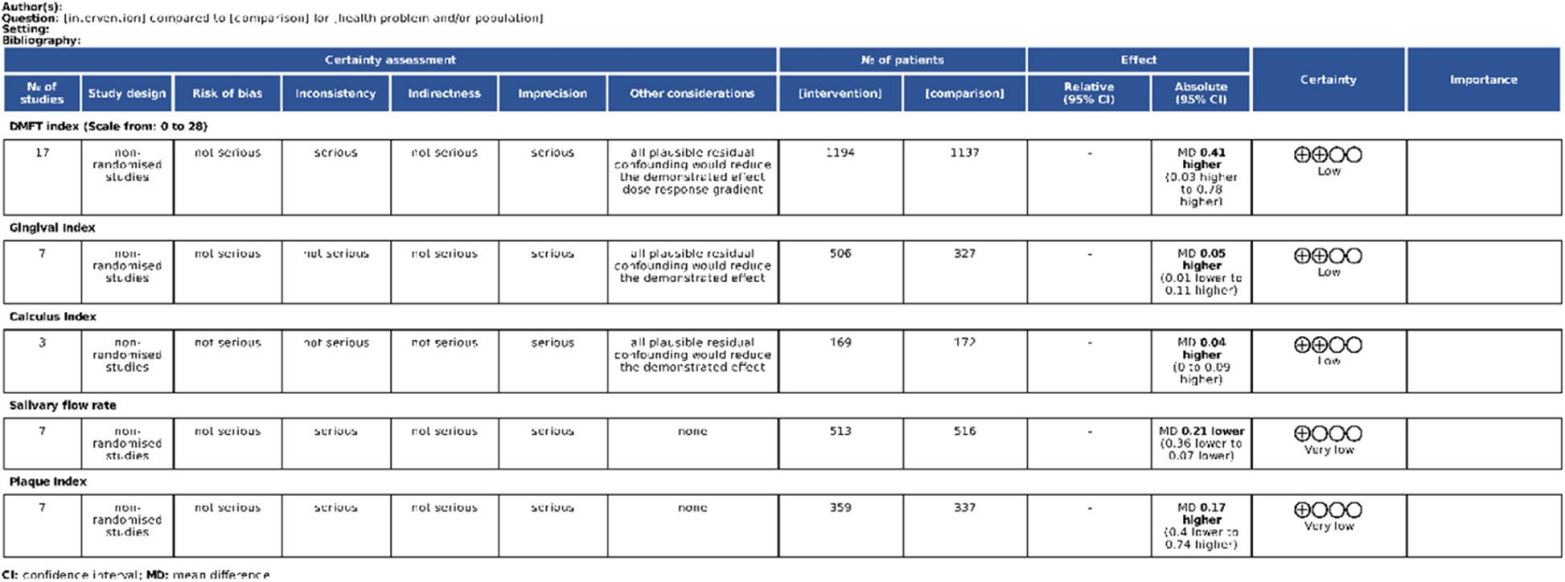
Assessment of the certainty of evidence with GRADE tool

Outcomes DMFT, GI, and CI have received low certainty of evidence, whereas outcomes salivary flow rate and PI have received very low certainty of evidence (Fig. 9).

## Discussion

The results of our meta-analysis regarding the pooled values of DMFT differences between patients with and without DM are in line with current state of the literature, however we only found a small difference between the groups, that is even though statistically significant, also clinically irrelevant, therefore a more complex approach is necessary to identify the connections more accurately [66].

The measurement of metabolic values in children holds significant importance as it facilitates early diagnosis and timely intervention. This approach enables full understanding of the potential consequences of DM, especially the effects of elevated blood glucose levels.

For instance, certain studies did not report statistically significant differences between the study and control groups. However, taking into account the differences in metabolic control, significant differences are found. For instance, Pachonski et al. reported no significant differences between DM and non-DM children concerning DMFT values. However, they observed statistically significant differences between PGC and non-DM children. [50]. Differences in metabolic control within the populations could give an explanation for some of the differences between the included studies, that may be responsible for some of the between study heterogeneity.

The most recent meta-analysis in the topic have found similar results regarding the differences in pooled DMFT values, however it did not investigate the effect of different glycemic controls on DMFT values [20]. Therefore, this meta-analysis sought to fill this gap in the literature.

The study of Elheeny et al. did not group the children with DM according to their quality of metabolic control, despite that, the study can be informative in this aspect. The frequency of children with PGC was higher in the age group between 8 and 10, than 11 and 14 with percentages of 93,6% and 76,3%, respectively – which means, especially for the early adolescent group, that they basically examined children with poorly controlled DM. They found significantly higher caries scores in both of these age groups [45]. However, in some cases, even when they examined more children with PGC, they did not observe significant differences between the study groups and the control groups. In the study of Lai, 70.6% of the children living with DM had PGC; in the study of Sadeghi, 40% of the DM children had PGC; and in the study of Mesaro S., 66.7% of the study group had high HbA1c values [40, 59, 60]. However, these percentages are significantly lower than those previously mentioned.

Most of the articles showed no significant differences between the study groups and the control groups. Some even reported significantly better DMFT indexes in children living with DM type I [36, 37, 39, 58, 63]. There could be several factors behind these results. Lower caries prevalence corresponds with the lower plaque scores, which could mean that DM children have better oral health routines than healthy children [37]. We have found no significant difference in PI between children with and without DM, that is in line with other studies [67]. Dental plaque is the strongest risk factor of developing caries, and the fact that PI is similar in the two population elevates the evidence of the impact of DM on caries risk [68]. It is said that children living with type 1 DM represent a more health-conscious and motivated group of society, due to the fact that these children are diagnosed with a metabolic disease at a young age and their parents are willing to cooperate with doctors and dentists to provide better life circumstances for their children [64]. This is confirmed in few studies; children with GGC had the best results not only compared to children with PGC but also to healthy controls [48, 50]. Lai et al. have reported that children with GGC are counted as patients with lower caries risk in contrast to children living with PGC. They did not observe a significant difference between the study and the control group, but there were significantly more caries-free children in the GGC group compared to the PGC group, and there was a statistically significant difference concerning many cariogenic bacteria [60].

Another reason for the outstanding DMFT values of DM patients are their strict, sucrose-restricted diet and frequent monitoring, which might answer the question of why children with GGC represent the lowest DMFT values [37, 48].

Furthermore, an important factor that could influence the results is the selection of patients in each group. For example, in the study of Iscan et al. 2020, control patients were children who sought treatment at the faculty, which could be a reason for an elevated value of DMFT score among them [54]. In another case, data of children with DM were collected at events organized to promote health-conscious lifestyles. Therefore, it may not represent the average DM population, hence parents that bring their children to such events are usually more health-conscious [64].

There is already evidence in the literature, that poor glycemic control in patients with type 2 DM elevates the risk of caries, periodontitis and peri-implantitis, however there were no previous analysis in the matter that investigated children with type 1 DM [69–71]. In order to fill this gap, we conducted the necessary analyses and found statistically significant, and clinically relevant differences between GGC and PGC children.

To have good glycemic control, it is essential to attend regular meetings with a diabetologist, who helps with motivation, cooperation, and education of health. Therefore, when examining the effects of DM, not only the presence of the illness is the most relevant factor, but the quality of metabolic control. In a few studies, the children living with type 1 DM had better parameters than the controls [36, 37, 39, 58, 63]. In other cases, only the children with GGC had better scores [60]. There was not a single case where children with PGC had better oral health parameters than controls or the GGC group.

We have found significantly lower salivary flow rate in children with DM, that could also provide a possible explanation for higher caries indices, that is in line with other studies conducted in the topic [72]. There was no article showing significantly better salivary flow rate in the DM group compared to the control groups’ scores. Pappa et al. examined not only the measurable salivary flow rate but the subjective feeling of xerostomia as well. Although they did not find a significant difference between the healthy and the DM groups, they found statistically significantly more children living with PGC suffering from xerostomia and lower salivary flow rate [48]. Children with GGC did not have significantly lower flow rates than the control patients; however, they reported xerostomia more often. According to Pappa et al., that could be a consequence of the frequent changes in blood sugar levels [48].

Also, we have found similar results regarding GI and CI parameters, that are closely connected with dental plaque induced inflammation, that further strengthens the connections of DM and caries [73, 74]. However, in the study of Babatzia et al., they found elevated amounts of plaque in the group of PGC children, there were no significantly higher GI index associated with it [47]. Additionally, some studies did not find significantly different values in CI either. However, it is important to note, that the formation of calculus and the induction of gingival inflammation could be affected by individual characteristics as well, not only the presence or absence of DM and dental plaque [40].

According to the results of our analysis, it is possible to conclude that PGC leads to higher prevalence of caries. There are many tools that enable dentists to measure their patients HbA1c levels without blood taking, pain, and with a relatively good cost- and time-efficient method, in the dental office [75]. Therefore, we suggest HbA1c measurements in the dental office for patients with DM, to check their quality of glycemic control, and to suggest diabetologist consultation when poor control is found.

Due to the nature of our research question, we could only include observational studies. Therefore, our certainty in our evidence is limited. Some included studies have not used the same indexes to report on periodontal condition, so it was not possible to include them in the quantitative analysis. The results for the meta-analysis have shown very high heterogeneity, which affects the certainty of the evidence. The strength of our study is, that to the best of our knowledge, there is no up-to-date analysis in the available literature on the topic that also investigates the impact of glycemic control on caries and periodontal outcomes. Hence, we could provide important insight in the topic.

According to our results, our implication for practice is that HbA1c measurements are highly advised among children with DM to screen for poor glycemic control and to prevent any possible further damage on oral and systemic health. The strive for good glycemic control, by improving patient compliance and encouraging good cooperation with diabetologists and dentists would benefit the oral and systemic health of children with type 1 DM.

Furthermore, we highly suggest more studies with rigorous protocols to compare children with different qualities of glycemic control according to their HbA1c levels to non-DM children, with cohorts matched for oral hygiene values.

## Conclusion

Children living with poorly controlled type 1 DM have higher DMFT values, while well-controlled children have comparable or better DMFT values to children with no DM. Chairside HbA1c measurement is highly suggested at dental checkups in order to identify underlying DM and verify the quality of glycemic control with close cooperation with diabetologist specialists.

## Data Availability

The datasets used in this study can be found in the full-text articles included in the systematic review and meta-analysis.
